# Epigenetic age acceleration is associated with cardiometabolic risk factors and clinical cardiovascular disease risk scores in African Americans

**DOI:** 10.1186/s13148-021-01035-3

**Published:** 2021-03-16

**Authors:** Farah Ammous, Wei Zhao, Scott M. Ratliff, Thomas H. Mosley, Lawrence F. Bielak, Xiang Zhou, Patricia A. Peyser, Sharon L. R. Kardia, Jennifer A. Smith

**Affiliations:** 1 Department of Epidemiology, School of Public Health, University of Michigan, Ann Arbor, MI USA; 2 Memory Impairment and Neurodegenerative Dementia (MIND) Center, University of Mississippi Medical Center, Jackson, MS USA; 3 Department of Biostatistics, School of Public Health, University of Michigan, Ann Arbor, MI USA; 4 Survey Research Center, Institute for Social Research, University of Michigan, Ann Arbor, MI USA

**Keywords:** Age acceleration, DNA methylation, Epigenetic age, Cardiovascular disease, Clinical risk scores, Cardiometabolic risk factors

## Abstract

**Background:**

Cardiovascular disease (CVD) is the leading cause of mortality among US adults. African Americans have higher burden of CVD morbidity and mortality compared to any other racial group. Identifying biomarkers for clinical risk prediction of CVD offers an opportunity for precision prevention and earlier intervention.

**Results:**

Using linear mixed models, we investigated the cross-sectional association between four measures of epigenetic age acceleration (intrinsic (IEAA), extrinsic (EEAA), PhenoAge (PhenoAA), and GrimAge (GrimAA)) and ten cardiometabolic markers of hypertension, insulin resistance, and dyslipidemia in 1,100 primarily hypertensive African Americans from sibships in the Genetic Epidemiology Network of Arteriopathy (GENOA). We then assessed the association between epigenetic age acceleration and time to self-reported incident CVD using frailty hazard models and investigated CVD risk prediction improvement compared to models with clinical risk scores (Framingham risk score (FRS) and the atherosclerotic cardiovascular disease (ASCVD) risk equation). After adjusting for sex and chronological age, increased epigenetic age acceleration was associated with higher systolic blood pressure (IEAA), higher pulse pressure (EEAA and GrimAA), higher fasting glucose (PhenoAA and GrimAA), higher fasting insulin (EEAA), lower low density cholesterol (GrimAA), and higher triglycerides (GrimAA). A five-year increase in GrimAA was associated with CVD incidence with a hazard ratio of 1.54 (95% CI 1.22–2.01) and remained significant after adjusting for CVD risk factors. The addition of GrimAA to risk score models improved model fit using likelihood ratio tests (*P* = 0.013 for FRS and *P* = 0.008 for ASCVD), but did not improve C statistics (*P* > 0.05). Net reclassification index (NRI) showed small but significant improvement in reassignment of risk categories with the addition of GrimAA to FRS (NRI: 0.055, 95% CI 0.040–0.071) and the ASCVD equation (NRI: 0.029, 95% CI 0.006–0.064).

**Conclusions:**

Epigenetic age acceleration measures are associated with traditional CVD risk factors in an African-American cohort with a high prevalence of hypertension. GrimAA was associated with CVD incidence and slightly improved prediction of CVD events over clinical risk scores.

**Supplementary Information:**

The online version contains supplementary material available at 10.1186/s13148-021-01035-3.

## Background

Cardiovascular disease (CVD) is the leading cause of mortality among US adults [1]. African Americans have the highest CVD morbidity and mortality burden, a trend which has been consistent over the last few decades [2]. Underlying this higher CVD prevalence is a greater burden of a number of risk factors, including hypertension, type 2 diabetes, and obesity [3–5]. Yet a focus on established risk factors and their management has failed to fully reduce the excess CVD burden among African Americans. Identification of novel biomarkers that go beyond traditional ones may help better identify at-risk individuals, advance precision medicine, and inform efforts to reduce CVD burden.

Epigenetic aging, based on DNA methylation (DNAm) at CpG dinucleotides, is a novel measure of biological aging that offers the opportunity to identify molecular markers of disease risk. The first generation of epigenetic aging measures, the HorvathAge [6] and HannumAge [7] epigenetic clocks, were trained on chronological age and are estimated based on 363 and 71 CpG sites selected using elastic net regression modeling, respectively. HorvathAge was trained using multi-tissue samples from children and adults, while HannumAge was trained using a single tissue (whole blood) from adults. Modified versions of these two measures were later derived to account for confounding by blood cell composition: intrinsic epigenetic age acceleration (IEAA) based on HorvathAge explicitly adjusts for blood cell composition, and extrinsic epigenetic age acceleration (EEAA) based on the HannumAge is a composite measure that includes a weighted average of cell counts known to vary strongly with age [8]. PhenoAge, a more recent measure based on whole blood from adults, was estimated using 513 CpG sites and was trained on a composite clinical measure of phenotypic age that is based on chronological age and nine biomarkers including albumin, creatinine, serum glucose, and white blood cell counts [9]. The biomarkers were selected for their association with the hazard of mortality using a Cox penalized regression model. GrimAge is another recent measure constructed based on the linear combination of 1030 CpG sites that represent DNAm-based surrogate measures for a number of plasma proteins and smoking pack-years [10]. Like PhenoAge, it is based on whole blood from adults. In addition to chronological age, both PhenoAge and GrimAge account for physiological dysfunction among individuals of the same chronological age in their selection of CpGs. For each of these measures, epigenetic age *acceleration* is defined as the discrepancy between epigenetic age and chronological age. These four epigenetic age acceleration measures are hypothesized to be capturing different aspects of aging and are based mostly on unique CpG sites [11].

A growing body of literature has examined the association between epigenetic age acceleration and CVD and its risk factors, such as blood pressure and lipids, but the overall evidence remains inconclusive likely due to heterogeneity in study design, the specific outcomes examined, and the epigenetic aging measures used [12–21]. PhenoAge and GrimAge are more recently developed measures, and so validation of their associations and comparisons to the first-generation measures are in early stages. Two recent studies in participants of European ancestry show that GrimAA outperforms other measures in its association with CVD incidence after adjusting for CVD risk factors [21, 22], and additional studies report similar findings with all-cause mortality [21–24]. Yet it is unclear whether epigenetic age acceleration measures could be used to improve CVD prediction in a clinical setting.

In this study, we investigated the relationship between four epigenetic age acceleration measures and ten cardiometabolic markers of hypertension, insulin resistance, and dyslipidemia in 1,100 primarily hypertensive African Americans in the Genetic Epidemiology Network of Arteriopathy (GENOA) study. We additionally assessed the association between four epigenetic age acceleration measures and incident CVD. Finally, we examined whether epigenetic age acceleration measures can improve the predictive accuracy of two clinically-used CVD risk scores: the Framingham risk score (FRS) [25] and the more recently developed atherosclerotic cardiovascular disease (ASCVD) risk equation [26].

## Results

### Descriptive statistics

Baseline characteristics of the participants are shown in Table 1. The 1,100 participants from 530 sibships had a mean age of 57.1 years, and 71% were women. About 60% were never smokers and mean alcohol consumption was 0.66 drinks per week. About 70% of the participants had hypertension and 20% had type 2 diabetes at baseline. At baseline, 91 participants had prevalent CVD and another 72 developed incident CVD over 8,161 person-years of follow-up. The mean Framingham risk score (FRS) was 14.4% and the mean of the atherosclerotic cardiovascular disease (ASCVD) risk equation was 11.6%. FRS and ASCVD were positively and significantly correlated (r = 0.94, *P* = 2.2 × 10^–16^). Additional file 1: Fig. 1 shows the scatterplots for each of the DNAm age measures with chronological age. As previously reported, all of the DNAm age measures were strongly and significantly correlated with chronological age (all r > 0.8, Additional file 1: Table 1) [27]. The means of the age acceleration measures ranged between 0.11 years for GrimAA and 0.38 years for PhenoAA. The acceleration measures were not strongly correlated with each other (r range 0.19–0.50), nor where they correlated with chronological age (Additional file 1: Table 1, Additional file 1: Fig. 2**).**

**Table 1:** Descriptive characteristics of GENOA African Americans (N = 1,100)

	Mean (SD) or N (%)
Female	781 (71.0%)
Age (years)	57.1 (10.6)
Education (years)	12.3 (3.5)
Smoking status	
Never	666 (60.5%)
Former	255 (23.2%)
Current	179 (16.3%)
Alcohol consumption (drinks/week)	0.66 (2.6)
BMI (kg/m^2^)	31.20 (6.6)
Type 2 diabetes	216 (19.6%)
Anti-hypertensive medication use	649 (59.0%)
Hypertension	771 (70.1%)
Epigenetic age acceleration	
IEAA (years) (N = 1099)	0.15 (4.8)
EEAA (years)	0.27 (5.9)
PhenoAA (years) (N = 1099)	0.38 (7.2)
GrimAA (years) (N = 1099)	0.11 (5.0)
Cardiometabolic parameters	
Systolic blood pressure (mmHg)	133.8 (21.6)
Diastolic blood pressure (mmHg)	77.7 (11.9)
Mean arterial pulse pressure (mmHg)	96.4 (13.5)
Pulse pressure (mmHg)	56.2 (17.7)
Glucose (mg/dl) (N = 883)	109.2 (42.1)
Insulin (mIU/l) (N = 882)	11.5 (13.4)
Total cholesterol (mg/dl) (N = 1098)	204.3 (45.2)
HDL-C (mg/dl)	55.2 (17.9)
LDL-C (mg/dl) (N = 1076)	120.8 (41.5)
Triglycerides (mg/dl) (N = 1099)	146.0 (82.1)
CVD 10-year risk scores	
Framingham risk score (%) (N = 945)	14.4 (12.7)
ASCVD risk equation (%) (N = 988)	11.6 (11.1)
Prevalent CVD at baseline	91 (8.3%)
Incident CVD at follow-up	72 (8.8 per 1000 person-years)

Cardiovascular disease (CVD) was defined as self-reported myocardial infarction, coronary artery revascularization, cerebrovascular events, or surgical carotid artery revascularization

SD, standard deviation; IEAA, intrinsic epigenetic age acceleration; EEAA, extrinsic epigenetic age acceleration; BMI, body mass index; HDL-C, high density lipoprotein; LDL-C, low density lipoprotein; CVD, cardiovascular disease; ASCVD, Atherosclerotic cardiovascular disease

^†^Total N = 991 at Phase II and N = 496 at Phase III with DNAm measures

### Association between epigenetic age acceleration and cardiometabolic risk factors

Table 2 shows the regression results from linear mixed models for the univariate associations between the epigenetic age acceleration measures and cardiometabolic risk factors with beta coefficients for 1-year increase in epigenetic age acceleration after adjusting for age, sex, and familial relatedness. Effect sizes are also reported below per 5-year increase, which is equivalent to approximately one standard deviation of the epigenetic acceleration measures. At *P* < 0.05, IEAA, EEAA, and PhenoAA were each associated with four cardiometabolic risk factors, while GrimAA was associated with five.

**Table 2:** Association between epigenetic age acceleration and cardiometabolic risk factors in GENOA African Americans

Cardiometabolic risk factor (outcome)	IEAA			EEAA			PhenoAA			GrimAA		
	β	95% CI	*P* value	β	95% CI	*P* value	β	95% CI	*P* value	β	95% CI	*P* value
SBP	**0.37**	**0.109, 0.627**	**0.005**^**†**^	**0.27**	**0.055, 0.485**	**0.014**	**0.22**	**0.050, 0.395**	**0.012**	**0.36**	**0.093, 0.627**	**0.008**
DBP	**0.16**	**0.009, 0.303**	**0.037**	0.04	− 0.083, 0.160	0.534	0.07	− 0.033, 0.163	0.195	0.02	− 0.133, 0.170	0.809
MAP	**0.23**	**0.056, 0.393**	**0.009**	0.11	− 0.026, 0.253	0.112	**0.12**	**0.004 0.229**	**0.042**	0.13	− 0.042, 0.305	0.138
PP	**0.22**	**0.026, 0.414**	**0.027**	**0.24**	**0.082, 0.403**	**0.003**^**†**^	**0.16**	**0.033, 0.293**	**0.014**	**0.35**	**0.145, 0.544**	**0.001**^**†**^
Log glucose	2 × 10^–3^	− 0.002, 0.006	0.280	**3 × 10**^**–3**^	**0, 0.007**	**0.049**	**4 × 10**^**–3**^	**0.001, 0.007**	**0.004**^**†**^	**8 × 10**^**–3**^	**0.003, 0.012**	**4.0 × 10**^**–4†**^
Log insulin	2 × 10^–3^	− 0.008, 0.011	0.724	**0.01**	**0.005, 0.020**	**0.002**^**†**^	0.01	0, 0.012	0.056	2 × 10^–3^	− 0.008, 0.011	0.722
Total cholesterol	− 0.22	− 0.778, 0.332	0.430	− 0.17	− 0.627, 0.291	0.473	− 0.06	− 0.431, 0.309	0.748	− 0.49	− 1.057, 0.080	0.092
Log HDL-C	− 3 × 10^–4^	− 0.004, 0.003	0.855	− 1 × 10^–3^	− 0.004, 0.002	0.475	1 × 10^–3^	− 0.001, 0.003	0.444	− 2 × 10^–3^	− 0.005, 0.002	0.328
LDL-C	− 0.38	− 0.894, 0.144	0.157	− 0.15	− 0.579, 0.281	0.498	− 0.23	− 0.576, 0.120	0.199	− **0.77**	− **1.303, -0.236**	**0.005**^**†**^
Log triglycerides	3 × 10^–3^	− 0.002, 0.009	0.214	1 × 10^–3^	− 0.004, 0.005	0.758	3 × 10^–3^	− 0.001, 0.007	0.105	**0.01**	**0.006, 0.017**	**1.05 × 10**^**–4†**^

Models are adjusted for age, sex, and familial relatedness

IEAA, intrinsic epigenetic age acceleration; EEAA, extrinsic epigenetic age acceleration; SBP, systolic blood pressure; DBP, diastolic blood pressure; MAP, mean arterial pressure; PP, pulse pressure; HDL-C, high density lipoprotein; LDL-C, low density lipoprotein

Effect sizes (β) correspond to the change in the cardiometabolic risk factor associated with a 1-year increase in the epigenetic age acceleration measure

Associations significant at *P* < 0.05 are shown in bold; ^†^Associations significant at Bonferroni adjusted *P* < 0.005

IEAA was associated with higher systolic blood pressure (SBP), and both EEAA and GrimAA were associated with higher pulse pressure after accounting for multiple testing. A 5-year increase in IEAA was associated with an approximately 1.85 mmHg increase in SBP (95% CI 0.55–3.14). For EEAA and GrimAA, a 5-year increase was associated with a 1.20 mmHg (95% CI 0.41–2.0) and a 1.75 mmHg (95% CI 0.73–2.72) increase in pulse pressure, respectively.

GrimAA was associated with higher fasting glucose levels and EEAA was associated with higher fasting insulin levels after accounting for multiple testing. A 5-year increase in GrimAA was associated with a 4.08% increase (95% CI 1.51–6.18%) in glucose levels. EEAA was the only measure associated with insulin, where a 5-year increase was associated with a 5.13% increase (95% CI 2.53–10.5%).

Only GrimAA was associated with any of the lipid traits examined after accounting for multiple testing. A 5-year increase in GrimAA was associated with a 3.85 mg/dl (95% CI − 6.50 to − 1.20) decrease in low density lipoprotein (LDL-C) and a 5.13% (95% CI 3.05–8.87%) increase in triglyceride levels. The associations between the lipid measures and GrimAA remained significant after excluding participants who were not fasting for at least 10 h (β = − 1.01, *P* = 0.001, N = 863 for LDL-C and β = 0.011, *P* = 0.001, N = 881 for triglycerides).

Additional file 1: Table 2 shows the adjusted linear mixed model regression results for associations significant at *P* < 0.05 from Table 2. Although some of the nominally significant associations fully attenuated after adjusting for education, smoking status, body mass index (BMI), and alcohol consumption (Model 2), all of the associations that were significant after multiple testing in the base model (Bonferroni-corrected *P* < 0.05) remained significant at *P* < 0.05. When we further adjusted PhenoAA and GrimAA associations for white blood cell counts (Model 3), all of the associations became less significant, and the associations between PhenoAA and glucose and GrimAA and pulse pressure were fully attenuated (*P* = 0.100 and *P* = 0.054, respectively). GrimAA, however, remained significantly associated with glucose, LDL-C, and triglycerides (*P* < 0.05).

### Epigenetic age acceleration associations with clinical cardiovascular risk scores and incident CVD

All epigenetic age acceleration measures were significantly associated with the FRS and the ASCVD risk equation, except for IEAA with FRS. The effect estimates from the linear mixed models were in the expected direction with increased biological aging associated with an increase in the predicted 10-year risk of CVD (Table 3**)**. The largest effect estimate was observed for GrimAA, where a 5-year increase in epigenetic age acceleration was associated with a 2.9% (95% CI 2.2–3.6%) and a 2.2% (95% CI 1.7–2.8%) increase in the 10-year CVD risk using the FRS and ASCVD equations, respectively.

**Table 3:** Association between epigenetic age acceleration and clinical CVD risk scores in GENOA African Americans

Epigenetic age acceleration (Predictor)	Framingham risk score (FRS) (N = 945)		Atherosclerotic cardiovascular disease equation (ASCVD) (N = 988)	
	β (95% CI)	*P* value	β (95% CI)	*P* value
IEAA	0.12 (− 0.02–0.26)	0.088	**0.17 (0.06–0.29)**	**0.004**
EEAA	**0.21 (0.10–0.32)**	**3.77 × 10**^**–4**^	**0.23 (0.13–0.33)**	**3.85 × 10**^**–6**^
PhenoAA	**0.15 (0.06–0.24)**	**0.001**	**0.18 (0.10–0.26)**	**4.78 × 10**^**–6**^
GrimAA	**0.58 (0.44–0.71)**	**4.53 × 10**^**–16**^	**0.44 (0.33–0.56)**	**2.38 × 10**^**–13**^

Models are adjusted for age, sex, and familial relatedness

IEAA, Intrinsic epigenetic age acceleration; EEAA, extrinsic epigenetic age acceleration

FRS and ASCVD are modeled as continuous predictors

Effect sizes (β) correspond to the change in predicted 10-year risk of CVD using the FRS or ASCVD risk equation associated with 1-year increase in the epigenetic age acceleration measure

Cardiovascular disease (CVD) was defined as self-reported myocardial infarction, coronary artery revascularization, cerebrovascular events, or surgical carotid artery revascularization

Associations significant at *P* < 0.05 are shown in bold

**Fig. 1:**
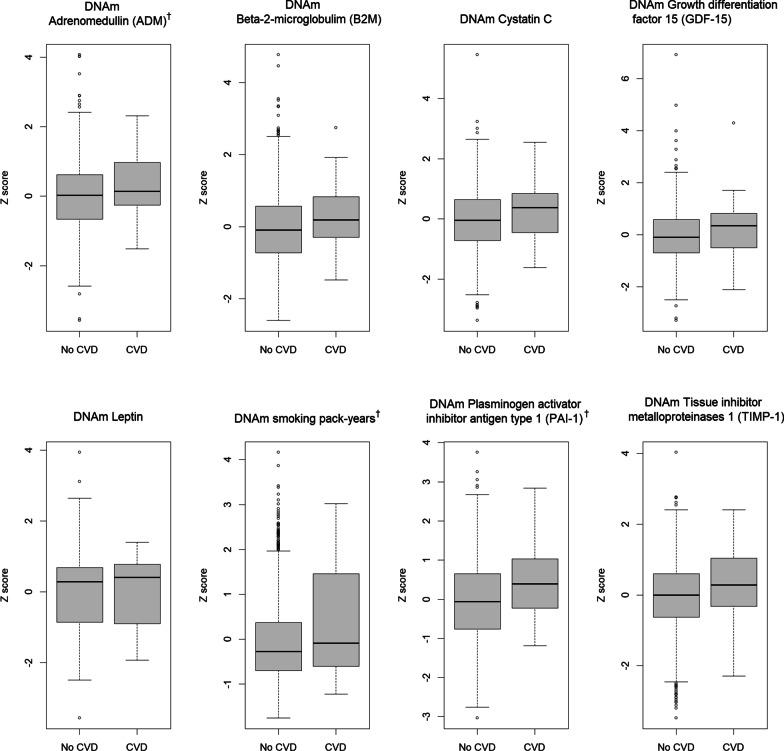
Boxplots of standardized GrimAge components by incident CVD status. CVD, cardiovascular disease. Cardiovascular disease (CVD) was defined as self-reported myocardial infarction, coronary artery revascularization, cerebrovascular events, or surgical carotid artery revascularization. ^†^ GrimAge components significantly associated with incident CVD in models adjusted for age, sex, and familial relatedness (*P* < 0.05)

When we examined whether epigenetic acceleration measures were associated with time to first CVD event, a similar trend emerged with GrimAA showing the only significant association with indicent CVD. Table 4 shows the hazard ratios (HR) and 95% confidence intervals estimated from Cox proportional hazards models with a frailty term for the associations of the four epigenetic age acceleration measures with incident CVD. A 5-year increase in GrimAA was associated with a HR of 1.54 (95% CI 1.22–2.01) in the base model (adjusted for age, sex, and familial relatedness). Further adjusting for traditional CVD risk factors (education, alcohol consumption, body mass index, total cholesterol, HDL-C, anti-hypertensive medication use, SBP, smoking status, and type 2 diabetes status) only slightly attenuated the association (HR per 5-year increase in GrimAA: 1.47, 95% CI 1.05–2.01, *P* = 0.024). Additionally adjusting for white blood cell counts did not attenuate the association (HR per 5-year increase in GrimAA: 1.54, 95% CI 1.10–2.19, *P* = 0.01). Findings were similar when time to CVD was modeled using interval censoring. Last, we examined the association between the individual components comprising GrimAge and incident CVD to identify components that may be driving the association between GrimAA and CVD or that outperform the overall GrimAA measure itself **(**Additional file 1: Table 3). Adrenomedullin (ADM), smoking pack-years, and plasminogen activator inhibitor antigen type 1 (PAI-1) were associated with incident CVD (*P* < 0.05) in the base model after further adjustment for white blood cell types, with HRs only slightly lower than that of GrimAA. Figure 1 shows the box plots of the standardized DNAm surrogate measures of the 7 plasma protein and smoking pack-years in GrimAge by incident CVD status. The means of ADM, smoking pack-years, and PAI-1 were higher among those with incident CVD.

**Table 4:** Incident CVD hazard ratios for epigenetic age acceleration in GENOA African Americans

Epigenetic age acceleration (predictor)	HR (95% CI)	*P* value
IEAA	0.97 (0.93–1.02)	0.300
EEAA	1.04 (1.00–1.08)	0.057
PhenoAA	1.00 (0.97–1.04)	0.810
GrimAA	**1.09 (1.04–1.15)**	**4.20 × 10**^**–4**^

Models are adjusted for age, sex and familial relatedness

CVD, cardiovascular disease; HR, hazard ratio; IEAA, intrinsic epigenetic age acceleration; EEAA, extrinsic epigenetic age acceleration

Hazard ratios correspond to the risk of a CVD event associated with a 1-year increase in the epigenetic age acceleration measure

Cardiovascular disease (CVD) was defined as self-reported myocardial infarction, coronary artery revascularization, cerebrovascular events, or surgical carotid artery revascularization

Associations significant at *P* < 0.05 are shown in bold

### Evaluating the performance of epigenetic age acceleration measures in CVD prediction

Likelihood ratio (LR) tests of nested models showed that GrimAA improved model fit when added to a model with age, sex, and FRS (HR per 1-year increase in GrimAA: 1.07, 95% CI 1.02–1.13, *P* for LR test of model fit = 0.013) or the ASCVD equation (HR per 1-year increase in GrimAA: 1.08, 95% CI 1.02–1.13, *P* for LR test = 0.008) (Table 5). None of the other age acceleration measures improved model fit.

**Table 5:** Incident CVD hazard ratios for GrimAA and clinical CVD risk scores in GENOA African Americans

Predictor	Adjusted HR (95% CI)			
	FRS only (N = 945)	ASCVD only (N = 988)	FRS + GrimAA (N = 945)	ASCVD + GrimAA (N = 988)
FRS	1.03 (1.02–1.05) *P* = 9.5 × 10^–6^	–	1.03 (1.01–1.05) *P* = 4.7 × 10^–4^	–
ASCVD	–	1.04 (1.02–1.06) *P* = 2.7 × 10^–5^	–	1.03 (1.01–1.05) *P* = 9.8 × 10^–4^
GrimAA	–	–	1.07 (1.02–1.13) *P* = 0.011	1.08 (1.02–1.13) *P* = 0.007

Models consisted of clinical risk scores with and without GrimAA. All models were adjusted for age, sex, and familial relatedness

Adjusted hazard ratios correspond to the risk of a CVD event associated with a 1-unit increase in the clinical risk score or the epigenetic age acceleration measure

Cardiovascular disease (CVD) was defined as self-reported myocardial infarction, coronary artery revascularization, cerebrovascular events, or surgical carotid artery revascularization

CVD, cardiovascular disease; HR, hazard ratio; FRS, Framingham risk score; ASCVD, atherosclerotic cardiovascular disease

Since GrimAA improved model fit, we next evaluated whether it could improve CVD risk prediction compared with the FRS and ASCVD risk equations using the C-statistic and the net reclassification index (NRI). The C-statistic is the probability that a randomly selected participant who experienced the CVD event will have a higher predicted probability of having the event compared to a randomly selected participant who did not experience the event. Table 6 shows the C-statistics for the performance of GrimAA in predicting incident CVD. The addition of GrimAA to a model with each risk score increased the C-statistic to 0.698 for FRS and to 0.685 for the ASCVD risk equation (all *P* > 0.05). Additional file 1: Fig. 3 shows the receiver operator characteristic (ROC) curves for the risk scores before and after adding GrimAA to the model.

**Table 6:** C-statistics evaluating the predictive performance of GrimAA on incident CVD in GENOA African Americans

Model	C-statistic	95% CI
N = 945		
Base model (age + sex)	0.595	0.525–0.664
Age + sex + GrimAA	0.643	0.576–0.709
Age + sex + FRS	0.687	0.624–0.749
Age + sex + FRS + GrimAA	0.698	0.637–0.759
N = 988		
Base model (age + sex)	0.588	0.521–0.656
Age + sex + GrimAA	0.636	0.571–0.701
Age + sex + ASCVD	0.670	0.606–0.728
Age + sex + ASCVD + GrimAA	0.685	0.625–0.746

The C statistics associated with a set of nested models for time to CVD events are shown. All models are adjusted for familial relatedness

Cardiovascular disease (CVD) was defined as self-reported myocardial infarction, coronary artery revascularization, cerebrovascular events, or surgical carotid artery revascularization

CVD, cardiovascular disease; FRS, Framingham risk score; ASCVD, atherosclerotic cardiovascular disease

Next, we compared the classification of CVD events with and without GrimAA using the NRI, an index of the net improvement in reassignment of the risk categories [28]. The FRS categorized 36.5% of the GENOA cohort as low risk (≤ 7.5%) while the ASCVD equation categorized 47.2% of the cohort as low risk. Net reclassification for CVD was small but significant with the addition of GrimAA to a model of age, sex, and FRS (NRI: 0.055, 95% CI 0.040–0.071, *P* < 0.0001), and with the addition of GrimAA to a model of age, sex, and the ASCVD equation (NRI: 0.029, 95% CI 0.006–0.064, *P* = 0.0011). Additional file 1: Fig. 4 shows the reclassification tables of predicted CVD based on the NRI for models with FRS or ASCVD and GrimAA. The improvement in risk prediction was driven by the classification of CVD nonevents as low risk.

When we excluded participants taking lipid-lowering statin medications, improvement in risk prediction in models with GrimAA was almost identical to that of the full sample. GrimAA remained associated with incident CVD after adding it to a base model with FRS and ASCVD (HR: 1.08 in both models, *P* = 0.004 and *P* = 0.003, respectively). As in the full sample, addition of GrimAA to a model with FRS or the ASCVD equation increased the C-statistics, but the increases were not significant at *P* < 0.05. The NRIs with the addition of GrimAA to the risk scores were also similar (NRI: 0.052 for FRS, NRI: 0.030 for the ASCVD equation).

## Discussion

In this study of primarily hypertensive African-American participants from GENOA, we showed that increased biological aging is associated with a worse cardiometabolic risk profile, although the associations with specific cardiometabolic risk factors varied across the age acceleration measures. All of the epigenetic acceleration measures were correlated with risk of CVD onset as modeled by clinical CVD risk scores (FRS and ASCVD equation). GrimAA outperformed IEAA, EEAA, and PhenoAA in predicting CVD incidence, and the association remained significant after adjusting for traditional CVD risk factors. The addition of GrimAA to FRS or ASCVD did not improve the C-statistics of CVD risk prediction; however, the NRIs showed small but significant improvement in the reassignment of risk categories.

Differences in the cardiometabolic risk factor and CVD incidence associations among the various epigenetic clocks may be attributed to a number of factors. IEAA, EEAA, and PhenoAA share only between 5 and 36 CpG sites [9]. Information on the CpGs included in GrimAge are not publicly available, so we cannot assess how many CpG sites this measure shares with the other three. In addition to differences in training algorithms (chronological age for IEAA and EEAA vs. aging correlates and outcomes for PhenoAA and GrimAA), the second generation of epigenetic measures (PhenoAA and GrimAA) were trained using longitudinal data [29]. This is particularly relevant for studies assessing their prediction of aging-related outcomes. The use of cross-sectional training data may have biased the algorithm as individuals with accelerated aging rates will have a higher morality burden and may have been selected out from the training samples [29, 30]. Nevertheless, an analysis of the transcriptional profiles of IEAA, EEAA, and PhenoAge shows that they have relatively similar transcriptional signatures [11].

Our study found cross-sectional associations between epigenetic age acceleration and a number of cardiometabolic risk factors. Out of the 10 cardiometabolic risk factors examined in the base model, GrimAA was associated with 4 of the measures, EEAA with 2 measures, and IEAA and PhenoAA with 1 measure after accounting for multiple testing. Some of the associations were unique to one specific cardiometabolic feature such as the association between GrimAA and lipid traits. For the significant associations between the acceleration measures and cardiometabolic risk factors, the effect directions were as expected with the exception of the association between GrimAA and LDL-C. Higher epigenetic acceleration, indicative of tissue aging faster than expected by chronological age, was associated with worsening outcomes as measured by cardiometabolic risk factors. Increased tissue aging in blood is accompanied by changes in cell-type composition [31]. However, the associations between GrimAA and cardiometabolic risk factors were not attenuated after adjusting for blood cell composition, with the exception of the association between GrimAA and pulse pressure. This suggests that the associations observed are not due to age-related changes in blood cell composition and that GrimAA is capturing cell-intrinsic properties or innate changes related to aging rather than changes in immune cell composition.

In our study, IEAA was associated with SBP, and both EEAA and GrimAA were associated with pulse pressure. Previous studies on the association between IEAA and EEAA and cardiometabolic measures show inconsistent findings [13, 20, 32, 33]. A previous study of IEAA and EEAA in the Women’s Health initiative (WHI) found no associations with systolic or diastolic blood pressure after adjusting for diet and metabolic syndrome symptoms [32]. However, in a smaller sample of African Americans from the Bogalusa Heart Study (N = 288), both IEAA and EEAA were associated with hypertension [20]. Another study of approximately 5,000 individuals from the Generation Scotland: Scottish Family Health Study found evidence of an association between EEAA and high blood pressure, but not IEAA [34]. A previous analysis in GENOA found no association between blood pressure measured at Phase II and IEAA or EEAA [35], although significant associations were detected in this study using concurrently measured blood pressure (Phase I). PhenoAA and GrimAA were more recently developed, so fewer studies have assessed their associations with cardiometabolic risk factors. However, in WHI, both PhenoAA and GrimAA were significantly correlated with SBP but not DBP [9, 10].

In this study, we also found evidence of associations between PhenoAA and GrimAA and glucose, and EEAA and insulin. GrimAA was the only measure associated with any of the lipid traits. In WHI, no associations between measures of insulin resistance and dyslipidemia (HDL-C and triglycerides) were detected with IEAA or EEAA, except for an association between EEAA and triglycerides (β = 0.004, *P* value = 0.04) [20]. However, other studies have reported an inverse association between fasting HDL-C levels and EEAA [13] and IEAA [34]. Cross-sectional examination of WHI revealed correlations between both PhenoAA and GrimAA and insulin, glucose, triglycerides, and HDL-C [9]. PhenoAA, but not GrimAA, was also correlated with LDL-C [10]. As in our study, GrimAA was associated with lower total cholesterol and LDL-C in a cross-sectional analyses of 709 individuals from the Lothian Birth Cohort [36]. Another study in the Methyl Epigenome Network Association and a Spanish cohort found significant correlations between GrimAA and glucose levels, HDL-C, and triglycerides [37].

In our analyses, higher GrimAA was the only measure associated with CVD incidence in GENOA African Americans independent of CVD risk factors. Adjustment for white blood cell counts did not attenuate the association. Neither EEAA nor IEAA were associated with incident coronary heart disease in WHI [20]. However, among Black participants from the Atherosclerosis Risk in Communities (ARIC) study, epigenetic age acceleration based on the Horvath and Hannum measures were both associated with increased hazard of fatal coronary heart disease (HR: 1.17, 95% CI 1.02–1.33 and HR: 1.22, 95% CI 1.04–1.44, respectively) [17]. A German case-cohort study reported an increase in the hazard of cardiovascular mortality associated with Horvath age acceleration [38], while a study in the Melbourne Collaborative Cohort found no association with the Horvath or Hannum measures [16]. Increased PhenoAA, but not HorvathAA, was also associated with increased risk of cardiovascular mortality in 500 males from the US Normative Aging Study [39].

Our findings are in line with the literature in cohorts of European ancestry, showing that GrimAA outperforms other measures in its association with CVD incidence [21, 22]. The effect size of GrimAA on CVD incidence appears to be remarkably similar across studies in European ancestry, and similar to our estimate in African Americans. Comparing the same four measures of epigenetic acceleration that we investigated, Hillary et al. found that over thirteen years of follow-up, GrimAA outperforms the other measures in terms of its association with incidence of heart disease (HR: 1.41, 95% CI 1.18–1.68, per 1 SD) [21]. Wang et al. found that a 1 SD increase in GrimAA was associated with elevated risk of myocardial infarction (HR: 1.44, 95% CI 1.16–1.79) and stroke (HR: 1.42, 95% CI 1.06–1.91) in a study of elderly participants from the Normative Ageing Study and the Cooperative Health Research in the Region of Augsburg (KORA) study [22].

To our knowledge, no previous study has assessed the performance of the epigenetic age acceleration measures in improving the predictive accuracy of clinical risk scores of CVD. GrimAA appears to marginally improve prediction of CVD events beyond traditional risk factors when assessed using NRI but not using changes in the area of the ROC curves. The gains in risk prediction were mostly due to down-classification of non-cases as low risk. More studies are needed to validate and replicate these findings. However, GrimAA may be a promising biomarker since it is a composite measure of multiple plasma proteins, some of which have been shown to be independent biomarkers that can improve CVD prediction [40–43]. Additionally, for some of the components of GrimAge (PAI-1, TIMP-1, and cystatin C), DNAm-based surrogates were found to outperform the observed biomarkers [10]. Lu et al. found that DNAm smoking pack-years was a more significant predictor of lifespan than self-reported smoking and that it predicted mortality even among non-smokers. This may be related to errors in self-reporting or because DNAm pack-years may capture intrinsic variation across individuals with lasting biological damage related to smoking [10].

Our study has a number of limitations. Our findings were based on self-reported events, with only the year of the event reported, which could be subject to recall bias. We also note that there was loss to follow-up between baseline and Phases II and III. Those lost to follow-up between baseline and Phase III were 1.63 years older and had 3.5 mmHg higher systolic blood pressure on average (*P* = 0.014). Additionally, individuals lost to follow-up had higher GrimAA, FRS, and ASCVD risk scores (all *P* < 0.05). This indicates that participants at greater risk of CVD events were more likely to be lost to follow-up. Another limitation is that although we adjusted for a number of important confounders, we lacked information on dietary data in GENOA African Americans. Finally, our sample is predominantly hypertensive and has an overrepresentation of women, so our findings may not be representative of other cohorts. A strength is that our study provides insights on the association between four different epigenetic aging measures and cardiometabolic risk factors and CVD in a relatively large cohort of older African Americans. In addition, we also explored improvement of CVD risk prediction by incorporating epigenetic aging measures in clinical risk equations and investigated potential molecular drivers of the observed associations.

## Conclusions

Epigenetic information is an important molecular readout of lifetime exposures. We have shown that epigenetic aging measures are associated with some cardiometabolic risk factors in this relatively large cohort of African Americans. GrimAge acceleration was the only measure associated with CVD incidence after adjusting for CVD risk factors. Further studies are needed to replicate and further investigate potential improvement of clinical risk prediction using GrimAge acceleration.

## Methods

### Study sample

Genetic Epidemiology Network of Arteriopathy (GENOA) is a community-based study in Rochester, MN and Jackson, MS that was established to identify genes influencing blood pressure and development of target organ disease [44]. In the first phase of GENOA (Phase I: 1996–2001), sibships with at least two adults with clinically diagnosed essential hypertension before age 60 were recruited, and all siblings in the sibship were invited to participate regardless of hypertension status [20]. Exclusion criteria included secondary hypertension, alcoholism or drug abuse, pregnancy, insulin-dependent diabetes mellitus, or active malignancy. In Phase I (i.e., baseline), a total of 1,583 non-Hispanic whites (Rochester, MN) and 1,854 African Americans (Jackson, MS) were enrolled. In the second phase (Phase II: 2001–2005), all participants were invited for a second examination. Eighty percent of African Americans (N = 1,482) and 75% of non-Hispanic whites (N = 1,213) from Phase 1 returned. At Phase III (2009–2011), 752 African Americans returned for a third examination. This study includes African-American participants who had their DNA methylation profiles measured in whole blood samples collected at Phase I. Demographic information, medical history, clinical characteristics, lifestyle factors, and blood samples were collected in each phase. Written informed consent was obtained from all participants and approval was granted by participating institutional review boards (University of Michigan, University of Mississippi Medical Center, and Mayo Clinic).

### DNA methylation and epigenetic age acceleration measures

The methods of DNA methylation processing have been previously described [45]. Briefly, genomic DNA from 1,106 African-American participants from Phase I and 304 from Phase II was extracted from stored peripheral blood leukocytes using AutoGen FlexStar (AutoGen, Holliston, MA). Sex mismatches and outliers were excluded using the shinyMethyl R package [46], probes with detection *P*-value < 10^–16^ were considered to be successfully detected [47] and both samples and probes that failed a detection rate of at least 10% were removed. The Noob method was used for individual background and dye-bias normalization [48] and the Regression on Correlated Probes method was used to adjust for the probe-type bias in the data [49]. White blood cell type proportions within the blood sample were estimated using Houseman’s method [50].

After quality control, a total of 1,100 samples from Phase I and 294 from Phase II were available for assessment of epigenetic age acceleration; however, only Phase I measures were included in this study. Methylation beta values were uploaded to the online Horvath epigenetic age calculator to calculate DNAm Age [51]. Four measures of epigenetic age (HannumAge, HorvathAge, PhenoAge, and GrimAge) were estimated for the current analysis. IEAA based on the Horvath measure are the regression residuals after adjusting for chronological age and blood cell count [6–8]. EEAA was calculated using the Hannum epigenetic age after incorporating weighted averages of three white blood cell types (naïve cytotoxic T cells, exhausted cytotoxic T cells, and plasmablasts) [7, 8]. PhenoAge and GrimAge are considered to be extrinsic measures of aging because they capture both cell intrinsic methylation changes as well as extracellular changes in blood cell composition [9, 10, 31]. We also estimated 7 DNAm based surrogate plasma proteins (adrenomedullin (ADM), beta-2-microglobulin, cystatin C, GDF-15, leptin, plasminogen activator inhibitor antigen type 1 (PAI-1), tissue inhibitor metalloproteinases 1 (TIMP-1)), and smoking pack-years that comprise GrimAge in order to identify individual components that may drive associations or that are more predictive than the overall measure itself [10].

### Cardiometabolic risk factors

Resting systolic blood pressure (SBP) and diastolic blood pressure (DBP) were measured by a random zero sphygmomanometer and a cuff appropriate for arm size. The second and third of three readings, after the participant sat for at least 5 min, were averaged for analysis [52]. Mean arterial pressure (MAP) was calculated as the weighted average of SBP and DBP (1/3*SBP + 2/3*DBP) and pulse pressure (PP) was calculated as the difference between SBP and DBP (SBP–DBP). Information on current anti-hypertensive medication use and lipid-lowering statin medication use were collected. Hypertension was defined as SBP ≥ 140 mmHg, DBP ≥ 90 mmHg, or anti-hypertensive medication use. Smoking was categorized as current, former, or never. Blood glucose and insulin levels were measured for participants fasting for at least 10 h. Serum total cholesterol, HDL-C, and triglycerides (TGs) were measured by standard enzymatic methods on a Hitcahi 911 Chemistry Analyzer (Roche Diagnostics, Indianapolis, IN). LDL-C was calculated using the Friedewald formula [LDL in mg/dl = TC–HDL-C–(TGs/5)] [53] and individuals with triglycerides levels ≥ 400 mg/dl were excluded from LDL-C association analysis. Type 2 diabetes was defined as fasting serum glucose concentration > 126 mg/dl or self-reported physician-diagnosed diabetes and current medication use (insulin or hypoglycemic agents). Educational attainment was based on self-reported years of education. Alcohol consumption was calculated as the number of drinks per week based on aggregated measurements of a variety of alcoholic drinks. Height was measured by stadiometer and weight by electronic balance and body mass index (BMI) was calculated as weight in kilograms divided by the square of height in meters.

### CVD events and risk scores

Framingham risk score (FRS), predicting the 10-year risk of a CVD (defined as coronary death, myocardial infarction, coronary insufficiency, angina, ischemic stroke, hemorrhagic stroke, transient ischemic attack, peripheral artery disease, and heart failure), was estimated using age, sex, total cholesterol, HDL-C, anti-hypertensive medication use, SBP, smoking status, and type 2 diabetes status after limiting the sample to individuals aged between 30 and 74 years (N = 945, events = 69, person-years = 7874.9) [25]. While FRS was developed in participants of European ancestry, the more recently described ASCVD risk equation [26] was developed using a pooled community-based population cohort with a higher proportion of African Americans and has been validated for prediction of clinical events in more race/ethnically diverse cohorts. The ASCVD risk equation is based on the same covariates as those in the FRS and it predicts the 10-year risk of developing a first ASCVD event, defined as nonfatal myocardial infarction or coronary heart disease death or fatal or nonfatal stroke. Using sex- and race-specific parameters, we estimated the ASCVD risk equation, after limiting the sample to those between the ages of 20–79 years (N = 988, events = 71, person-years = 8115.5). Risk scores were modeled as continuous variables and as categorical predictors where they were used to group participants into low risk (10-year risk ≤ 7.5%) or high risk (> 7.5%) groups [26].

Information about CVD, as reported by participants, was collected at baseline and at each subsequent follow-up phase. An event was defined as myocardial infarction, coronary revascularization (stenting, balloon angioplasty, or coronary artery bypass grafting), stroke (ischemic or hemorrhagic events), or surgical carotid artery revascularization. Participants only reported the year of CVD events. Time to CVD was modeled by setting the CVD event time at the mid-point of the year in which participants reported an event. For censored participants, follow-up time was set at the time point they were last interviewed.

### Statistical analysis

Outliers at more than 5 standard deviations from the mean were removed for the cardiometabolic outcomes and the epigenetic age acceleration measures. Glucose, insulin, HDL-C, and triglycerides were natural log-transformed as ln(measure + 1). Linear mixed models that account for familial relatedness were used to assess the cross-sectional univariate association between each epigenetic age acceleration measure (predictor) and each cardiometabolic risk factor (outcome) at Phase I. Base models were adjusted for age and sex (Model 1). In subsequent models, we additionally adjusted for education, smoking status, body mass index, and alcohol consumption (Model 2) and white blood cell counts for PhenoAA and GrimAA to assess confounding by changes in cell composition (Model 3). For LDL-C and triglycerides, we performed sensitivity analyses excluding participants who were not fasting for at least 10 h before the blood draw.

After excluding participants with baseline CVD events, associations with time to first CVD event (incident CVD) were assessed using Cox proportional hazards models, and hazard ratios (HR) and 95% confidence intervals were estimated. A simple random effects (frailty) term in the Cox model was included to take into account family structure [54]. We next adjusted for traditional CVD risk factors (age, sex, education, body mass index, alcohol consumption, total cholesterol, HDL-C, anti-hypertensive medication use, SBP, smoking status, and type 2 diabetes status). Finally, we adjusted for white blood cell counts. The proportional hazard assumption was evaluated using Schoenfeld residuals, and all models satisfied the assumption. As a sensitivity analysis, we additionally modeled time to CVD using interval censoring using the iceReg package [55].

Likelihood ratio (LR) testing of nested models (addition of epigenetic age acceleration to a base model with either FRS or ASCVD) was used to assess improvement in model fit. For measures with *P* < 0.05, we assessed improvement in risk prediction of incident CVD by adding the epigenetic age acceleration measure to the base model with the clinical risk scores. We assessed the improvement in CVD risk prediction using C-statistics computed from Cox proportional hazards models of time to CVD events and risk scores as continuous predictors [56]. We additionally used the net reclassification index (NRI) to assess net improvement in reassignment of the risk categories [28]. Categorized CVD risk scores were used in the base model and improvement in risk reassignment was then assessed after the addition of epigenetic acceleration measures. For this analysis, we also examined the associations excluding individuals taking lipid-lowering statin medications (N = 40).

Statistical tests were two-sided and a *P* value of < 0.05 was considered nominally associated. We also applied a Bonferroni threshold for statistical significance (0.05/10 = adjusted *P* < 0.005) to account for multiple testing in assessing the association between epigenetic acceleration measures and the 10 cardiometabolic traits. For NRI, bootstrapping (10,000 interations) was used to compute 95% confidence intervals, and an empirical *P* < 0.05 was considered significant. Analyses were performed using R (Version 4.0.2) [57] and the following packages: lme4 [58], survival [59, 60], nricens, and DescTools.

## Supplementary Information

**Additional file 1**. Supplementary material, including Supplemental Tables 1–3 and Supplemental Figures 1–4.

## Data Availability

For this analysis, genotype and phenotype data are from the Database of Genotypes and Phenotypes (dbGaP): phs001238.v2.p1. Methylation data is from the Gene Expression Omnibus (GEO): GSE157131. Due to IRB restriction, mapping of the sample IDs between genotype data (dbGaP) and DNA methylation data (GEO) cannot be provided publicly but are available upon written request to JAS and SLRK.

## Abbreviations

ADM: Adrenomedullin
ASCVD: Atherosclerotic cardiovascular disease
BMI: Body mass index
CVD: Cardiovascular disease
DBP: Diastolic blood pressure
DNAm: DNA methylation
EEAA: Extrinsic epigenetic age acceleration
FRS: Framingham risk score
GENOA: Genetic Epidemiology Network of Arteriopathy
HDL-C: High density lipoprotein cholesterol
IEAA: Intrinsic epigenetic age acceleration
LDL-C: Low density lipoprotein cholesterol
LR: Likelihood ratio
MAP: Mean arterial pressure
NRI: Net reclassification index
PAI-1: Plasminogen activator inhibitor antigen type 1
PP: Pulse pressure
ROC: Receiver operator characteristic
SBP: Systolic blood pressure
TC: Total cholesterol
TIMP-1: Tissue inhibitor metalloproteinases 1
TG: Triglycerides
US: United States
