# Supplementary material for: Epigenetic age acceleration is associated with cardiometabolic risk factors and clinical cardiovascular disease risk scores in African Americans
Source: Clin Epigenetics. 2021 Mar 16;13:55. doi: 10.1186/s13148-021-01035-3 (PMC7962278; doi:10.1186/s13148-021-01035-3)
Supplement: Supplementary file 1 — Additional file 1. Supplementary material, including Supplemental Tables 1–3 and Supplemental Figures 1–4. [file 13148_2021_1035_MOESM1_ESM.docx]

**Supplemental Table 1:** **Pearson correlations between age, DNA methylation age, and epigenetic age acceleration in GENOA African Americans**

|  |  | Age | HorvathAge | HannumAge | PhenoAge | GrimAge | IEAA | EEAA | PhenoAA |
| --- | --- | --- | --- | --- | --- | --- | --- | --- | --- |
| HorvathAge |  | 0.86*** |  |  |  |  |  |  |  |
| HannumAge |  | 0.90*** | 0.90*** |  |  |  |  |  |  |
| PhenoAge |  | 0.82*** | 0.84*** | 0.85*** |  |  |  |  |  |
| GrimAge |  | 0.85*** | 0.78*** | 0.81*** | 0.80*** |  |  |  |  |
| IEAA |  | 0.02 | 0.51*** | 0.24*** | 0.28*** | 0.12*** |  |  |  |
| EEAA |  | 0.00 | 0.28*** | 0.43*** | 0.30*** | 0.15*** | 0.43*** |  |  |
| PhenoAA |  | -0.01 | 0.23*** | 0.20*** | 0.56*** | 0.16*** | 0.44*** | 0.50*** |  |
| GrimAA |  | -0.02 | 0.07* | 0.07* | 0.17*** | 0.50*** | 0.19*** | 0.27*** | 0.32*** |

*** p < .0001; ** p < .01; * p < .05

Abbreviations: IEAA, intrinsic epigenetic age acceleration; EEAA, extrinsic epigenetic age acceleration.

**Supplemental Table 2: Adjusted associations between epigenetic age acceleration and cardiometabolic risk factors for associations with *P* < 0.05 in the base model (Model 1) in GENOA African Americans**

|  |  |  | **Model 2** | | |  | **Model 3** | | |
| --- | --- | --- | --- | --- | --- | --- | --- | --- | --- |
| **Cardiometabolic Risk Factor (Outcome)** | **Epigenetic Age Acceleration (Predictor)** |  | **β** | **95% CI** | ***P* value** |  | **β** | **95% CI** | ***P* value** |
| **SBP** | **IEAA** |  | **0.33** | **0.075, 0.594** | **0.012** |  |  |  |  |
|  | **EEAA** |  | **0.23** | **0.012, 0.443** | **0.039** |  |  |  |  |
|  | **PhenoAA** |  | 0.17 | -0.002, 0.347 | 0.053 |  | 0.14 | -0.047, 0.329 | 0.141 |
|  | **GrimAA** |  | 0.31 | -0.024, 0.638 | 0.069 |  | 0.29 | -0.070, 0.639 | 0.115 |
| **DBP** | **IEAA** |  | **0.16** | **0.010, 0.305** | **0.037** |  |  |  |  |
| **Mean arterial pressure** | **IEAA** |  | **0.22** | **0.0459, 0.385** | **0.013** |  |  |  |  |
|  | **PhenoAA** |  | 0.10 | -0.018, 0.210 | 0.100 |  | 0.10 | -0.019, 0.226 | 0.099 |
| **Pulse pressure** | **IEAA** |  | 0.18 | -0.010, 0.378 | 0.063 |  |  |  |  |
|  | **EEAA** |  | **0.20** | **0.043, 0.365** | **0.013** |  |  |  |  |
|  | **PhenoAA** |  | 0.12 | -0.011, 0.25 | 0.073 |  | 0.06 | -0.080, 0.200 | 0.403 |
|  | **GrimAA** |  | **0.34** | **0.095, 0.588** | **0.007** |  | 0.26 | -0.004, 0.523 | 0.054 |
| **Log glucose** | **EEAA** |  | 2 × 10^-3^ | -0.001, 0.006 | 0.137 |  |  |  |  |
|  | **PhenoAA** |  | **3 × 10^-3^** | **0, 0.005** | **0.047** |  | 2 × 10^-3^ | -0.001, 0.005 | 0.100 |
|  | **GrimAA** |  | **8 × 10^-3^** | **0.003, 0.013** | **0.001** |  | **8 × 10^-3^** | **0.003, 0.013** | **0.004** |
| **Log insulin** | **EEAA** |  | **0.010** | **0.003, 0.017** | **0.004** |  |  |  |  |
| **LDL-C** | **GrimAA** |  | **-1.05** | **-1.709, -0.383** | **0.002** |  | **-0.74** | **-1.444, -0.025** | **0.043** |
| **Log Triglycerides** | **GrimAA** |  | **0.01** | **0.001, 0.015** | **0.026** |  | **0.01** | **0.0002, 0.015** | **0.045** |

Abbreviations: IEAA, intrinsic epigenetic age acceleration; EEAA, extrinsic epigenetic age acceleration; SBP, systolic blood pressure; DBP, diastolic blood pressure; HDL-C, high density lipoprotein; LDL-C, low density lipoprotein.

Model 1 (base model) is adjusted for age, sex, and familial relatedness. Results for Model 1 are shown in Table 2. Only associations that were significant in Model 1 (*P*<0.05) were further evaluated and reported in this table.

Model 2 is adjusted for Model 1 and years of education, smoking status, body mass index, and alcohol consumption.

Model 3 is adjusted for Model 2 and white blood cell counts.

Effect sizes (β) correspond to the change in the cardiometabolic risk factor associated with a 1-year increase in the epigenetic age acceleration measure.

Associations significant at *P* < 0.05 are shown in bold.

**Supplemental Table 3: Incident CVD hazard ratios for GrimAge components in GENOA African Americans**

| GrimAge component  (Predictor) | HR (95% CI) | *P* value |
| --- | --- | --- |
| Adrenomedullin (ADM) | **1.45 (1.07 – 1.98)** | **0.017** |
| Beta-2-microglobulim (B2M) | 1.04 (0.77 – 1.41) | 0.780 |
| Cystatin C | 1.38 (0.95 – 1.99) | 0.089 |
| Growth differentiation factor 15 (GDF-15) | 1.21 (0.92 – 1.59) | 0.170 |
| Leptin | 1.38 (0.91 – 2.10) | 0.130 |
| Smoking pack-years | **1.36 (1.09 – 1.72)** | **0.0076** |
| Plasminogen activator inhibitor antigen type 1 (PAI-1) | **1.48 (1.17 – 1.87)** | **0.0012** |
| Tissue inhibitor metalloproteinases 1 (TIMP-1) | 1.59 (0.97 – 2.60) | 0.068 |

Abbreviations: CVD, cardiovascular disease; HR, hazard ratio.

Models adjusted for age, sex, white blood cell counts, and familial relatedness.

Hazard ratios significant at P < 0.05 are shown in bold font.

Hazard ratios correspond to the risk of a CVD event associated with a one-standard deviation increase in the GrimAge component.

Cardiovascular disease (CVD) was defined as self-reported myocardial infarction, coronary artery revascularization, cerebrovascular events, or surgical carotid artery revascularization.

Associations significant at *P* < 0.05 are shown in bold.

**Supplemental Figure 1: Scatterplots of DNA methylation age measures against chronological age in GENOA African Americans**


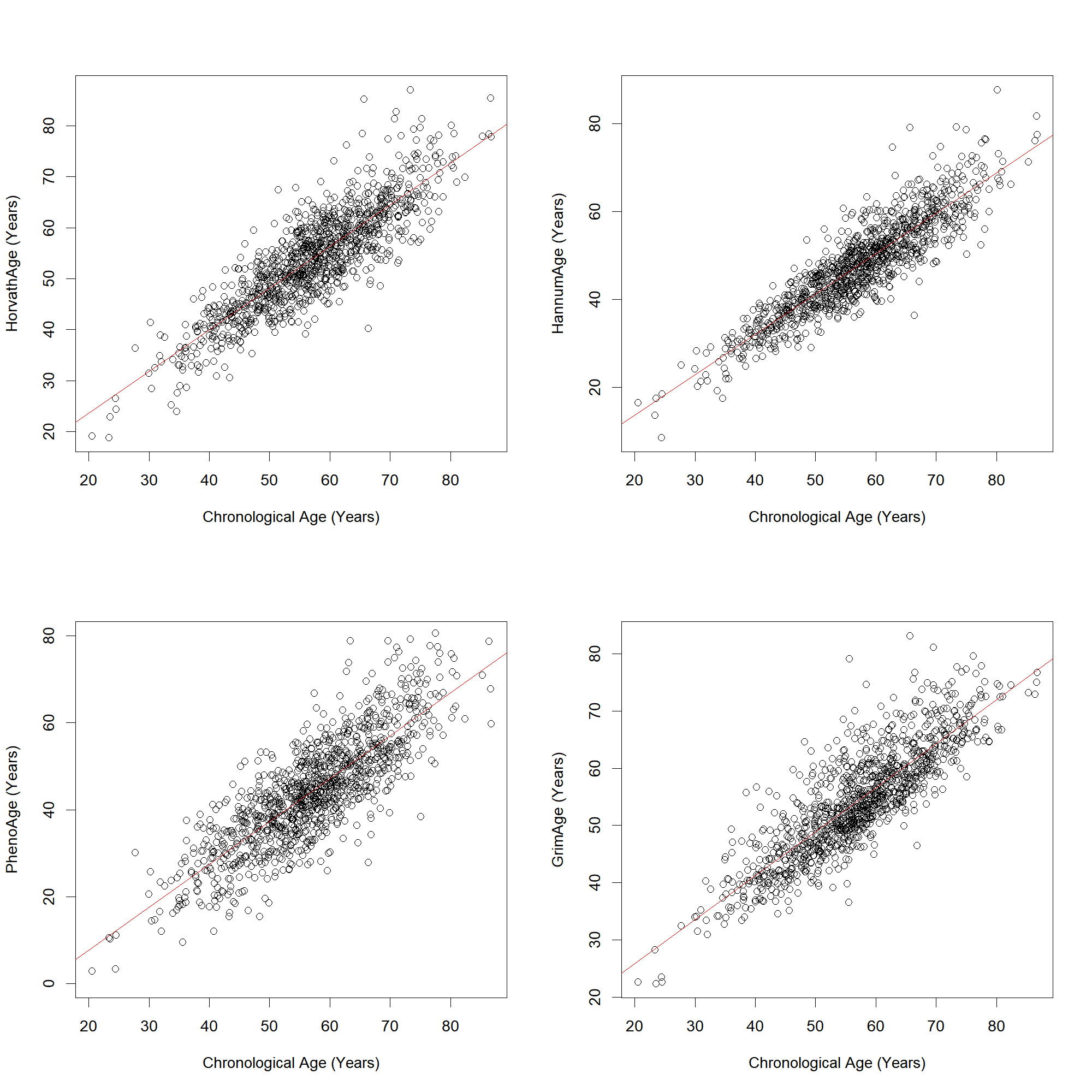


**Supplemental Figure 2: Scatterplots of epigenetic age acceleration measures against chronological age in GENOA African Americans**


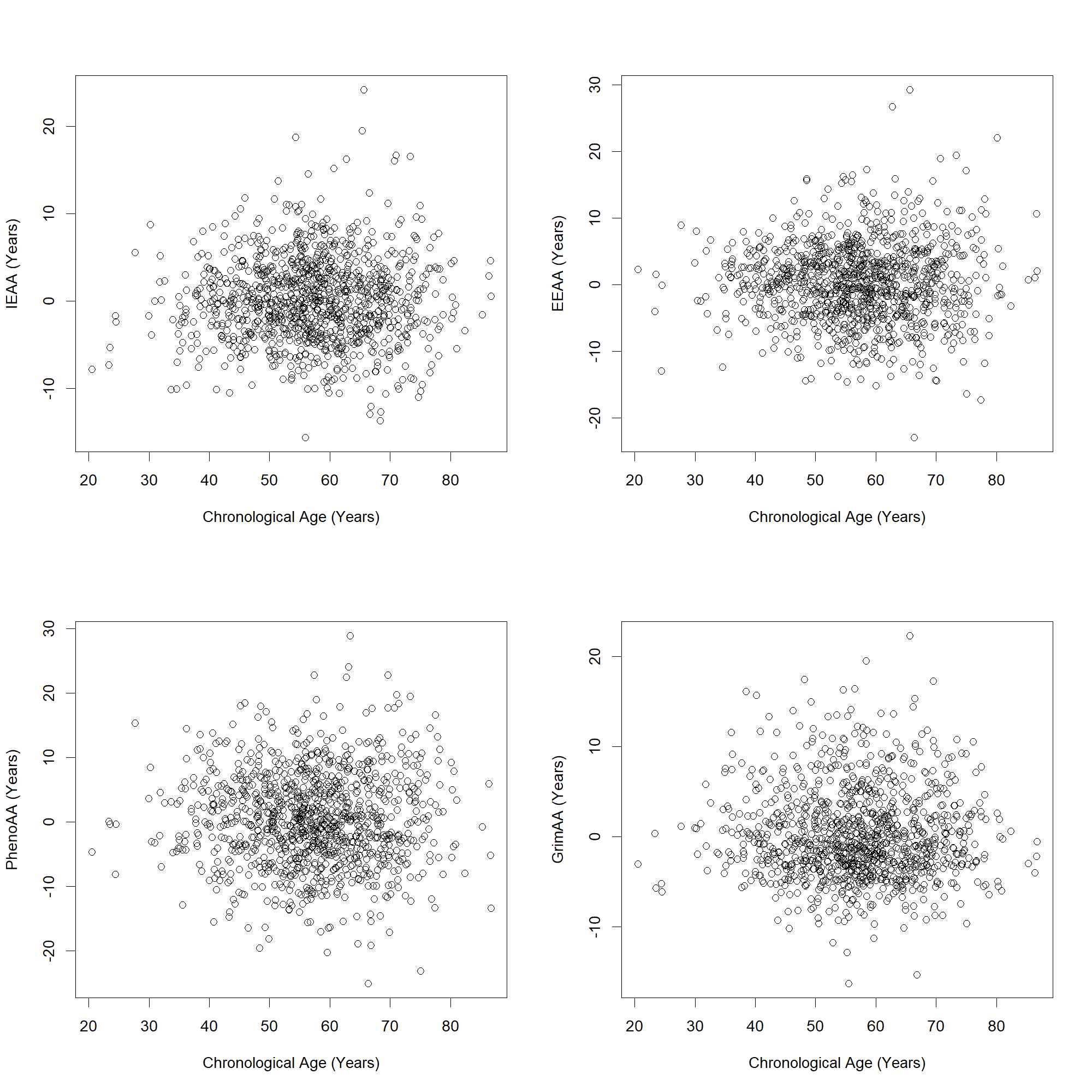


**Supplemental Figure 3: Receiver operator characteristic (ROC) curves for incident CVD in GENOA African Americans**

1. FRS vs. FRS + GrimAA (B) ASCVD vs. ASCVD + GrimAA


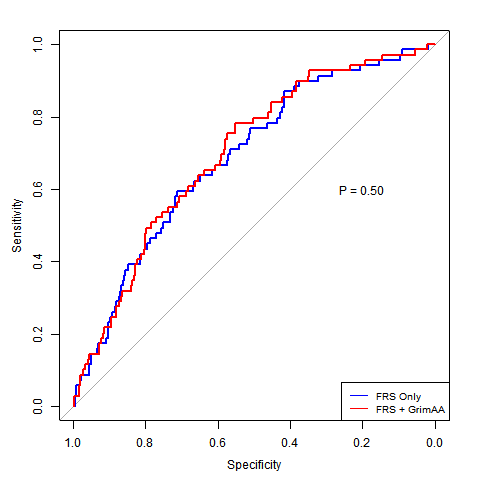

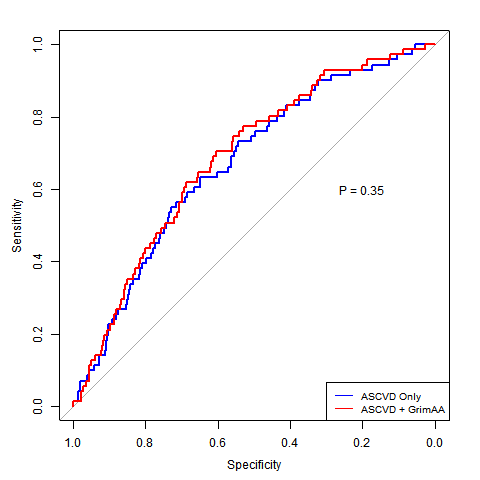


1. Receiver operator curves for time to CVD for models of Framingham risk score (blue line) vs. Framingham risk score + GrimAA (red line) (N = 945).
2. Receiver operator curves for time to CVD for models of ASCVD score (blue line) vs. ASCVD + GrimAA (red line) (N = 988).

Models are adjusted for age, sex and familial relatedness.

Cardiovascular disease (CVD) was defined as self-reported myocardial infarction, coronary artery revascularization, cerebrovascular events, or surgical carotid artery revascularization.

Abbreviations: CVD, cardiovascular disease; FRS, Framingham risk score; ASCVD, atherosclerotic cardiovascular disease.

**Supplemental Figure 4: Improvement in reclassification of incident CVD in GENOA African Americans**

1. FRS + GrimAA

|  |  | Standard CVD Model + GrimAA | | |
| --- | --- | --- | --- | --- |
|  | FRS  model | ≤ 7.5% | > 7.5% | Total no. (%) of participants |
| CVD Events | ≤ 7.5% | 7 | 0 | **7** |
|  | > 7.5% | 0 | 62 | **62** |
|  | Total no. (%) of participants | **7 (10.1)** | **62 (89.9)** | **69** |
| CVD Nonevents | ≤ 7.5% | 337 | 1 | **338** |
|  | > 7.5% | 49 | 489 | **538** |
|  | Total no. (%) of participants | **386 (44.1)** | **490 (55.9)** | **876** |

Columns and rows refer to categories of predicted risk using (A) FRS and GrimAA and (B) ASCVD and GrimAA. The counts in the cells represent the number of individuals assigned to the indicated risk category.

Blue shaded cells represent correctly reclassified individuals, and orange shaded cells represent incorrectly classified individuals.

NRI = P(up|case) – P(down|case) – P(up|noncase) + P(down|noncase)

In the model with FRS and GrimAA (A), 1 individual was incorrectly up-classified and 49 individuals were correctly down-classified (NRI = 49/876 – 1/876)

In the model with ASCVD and GrimAA (B), 1 individual was correctly-up classified. 9 individuals were incorrectly up-classified, and 23 individuals were correctly down-classified (NRI = 1/71 – 9/917 + 23/917)

Abbreviations: CVD, cardiovascular disease; FRS, Framingham risk score; ASCVD, atherosclerotic cardiovascular disease

Models are adjusted for age, sex and familial relatedness.

Cardiovascular disease (CVD) was defined as self-reported myocardial infarction, coronary artery revascularization, cerebrovascular events, or surgical carotid artery revascularization.

1. ASCVD + GrimAA

|  |  | Standard CVD Model + GrimAA | | |
| --- | --- | --- | --- | --- |
|  | ASCVD model | ≤ 7.5% | > 7.5% | Total no. (%) of participants |
| CVD Events | ≤ 7.5% | 14 | 1 | **15** |
|  | > 7.5% | 0 | 56 | **56** |
|  | Total no. (%) of participants | **14 (19.7)** | **57 (80.3)** | **71** |
| CVD Nonevents | ≤ 7.5% | 442 | 9 | **451** |
|  | > 7.5% | 23 | 443 | **466** |
|  | Total no. (%) of participants | **465 (49.7)** | **452 (50.3)** | **917** |
